# Personality traits of patients with multiple sclerosis and their correlation with anxiety and depression levels: A cross‐sectional case–control study

**DOI:** 10.1002/brb3.2596

**Published:** 2022-04-22

**Authors:** Amirali Ghahremani, Sahar Mosa Farkhani, Mahsa Baniasadi, Seyed Kaveh Hojjat, Hasan Namdar Ahmadabad, Davoud Salarbashi, Sepideh Elyasi, Najmeh Davoodian

**Affiliations:** ^1^ Department of Neurology North Khorasan University of Medical Sciences Bojnord Iran; ^2^ Department of Neurology North Khorasan University of Medical Sciences Bojnord Iran; ^3^ Department of Psychiatry, Division of Sleep Medicine, Psychiatry and Behavioral Sciences Research Center Mashhad University of Medical Sciences Mashhad Iran; ^4^ Department of Pathobiology and Laboratory Sciences North Khorasan University of Medical Sciences Bojnord Iran; ^5^ Nursing Research Center Gonabad University of Medical Sciences Gonabad Iran; ^6^ Department of Food Science and Nutrition School of Medicine Gonabad University of Medical Sciences Gonabad Iran; ^7^ Department of Clinical Pharmacy, Faculty of Pharmacy Mashhad University of Medical Sciences Mashhad Iran; ^8^ Infectious Diseases Research Centre Gonabad University of Medical Sciences Gonabad Iran

**Keywords:** anxiety level, depression level, multiple sclerosis, personality traits

## Abstract

**Objective:**

Multiple sclerosis is a chronic demyelinating disease of the central nervous system that can cause severe disability and impair the quality of life (QoL).

**Methods:**

In the current cross‐sectional, case–control study, we investigated personality traits, anxiety and depression levels, in 101 patients in the case group and 202 individuals as a control group. The personality traits of the participants were collected via the Neuroticism‐Extraversion‐Openness Five‐Factor Inventory (NEO‐FFI) questionnaire. We evaluated the level of anxiety and depression based on the Hospital Anxiety and Depression Scale questionnaire.

**Results:**

Our study showed in patients with disease duration above 1 year, the rates of agreement (29.78), anxiety (8.83), and depression level (6.39) were significantly higher than the control group (27.19, 6.47, and 4.97, respectively). Although patients with disease duration below 1 year showed a higher level of agreement and conscientiousness (29.65 and 34.35, respectively) than controls (26.6 and 30.86, respectively). The level of anxiety and depression in patients with a disability index above 4.5 was significantly higher than patients with a disability index below 1. Patients with a disability index below 1 showed a higher rate of extraversion and agreement and conscientiousness (31.47, 31.53, and 35.07, respectively) than controls (25.5, 26.23, and 3033, respectively). In addition, patients with a disability index above 4.5 showed a higher level of agreement (35.64), conscientiousness (35.5), anxiety (9.64), and depression (7.5) than controls (25.96, 30.71, 6.96, and 4.71, respectively).

**Conclusions:**

In conclusion, anxiety and depression levels were much higher among MS patients compared with controls and the severity of these conditions correlate with the score of the disability index. Therefore, a complete comprehension of these conditions by the neurologist could be vital in improving patients’ QoL and increasing compliance and adherence to pharmacological therapy.

## Graphical abstract

Anxiety and depression levels were much higher among MS patients compared with controls. The severity of these conditions correlates with the score of the disability index.

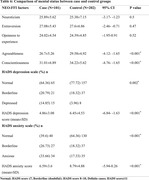

## 1 INTRODUCTION

Multiple sclerosis (MS) is a chronic demyelinating disease of the central nervous system that can cause severe disability and impairment in quality of life (QoL) (Goldenberg, 2012). This inflammatory disease is the most common cause of nontraumatic neurological disability among young and middle‐aged adults (Bizzoco et al., 2009).

MS patients suffer from various neuropsychiatric disturbances such as depression or cognitive impairment (Kurtzke, 1983). Psychiatric symptoms in MS have been known since late nineteenth century (Silveira et al., 2019). These symptoms were euphoria, mania, hallucinations, pathological laughing and weeping, and depression (Noy et al., 1995). These symptoms considerably interfere with daily activities, family relationships, and social and working life. These symptoms also disrupt emotion and well‐being and consequently reduce the QoL (Warren et al., 2001). The lifetime prevalence and incidence of major depression in MS is more than 50%, which is higher than the normal population as well as other neurological illnesses (Siegert & Abernethy, 2005). Unfortunately, it is often not detected and treated appropriately. It is also noteworthy that depression is an essential determinant of QoL in MS disease (D'Alisa et al., 2006). There is some evidence that depression in MS patients is correlated with neuropathology in the left anterior temporal/parietal region (Pandya et al., 2012).

Anxiety is also expected in MS patients. However, there is limited epidemiological evidence in this regard compared with depression (Kessler & Bromet, 2013). Some studies revealed that depression is the most significant factor related to anxiety in MS patients. Anxiety has been estimated to affect between 15.8 and 57% of the MS population (Karimi et al., 2020). In one study, half of the new patients and their parents had clinically high levels of anxiety and distress (Janssens et al., 2003).

Moreover, a contemporary report by the American Academy of Neurology announced that there is a possible relationship between antecedent stress and MS onset as well as MS exacerbation. Therefore, anxiety in MS patients not only affect the QoL but also exacerbate the disease (Goodin et al., 1999).

Early diagnosis of these clinical manifestations is vital in order to offer suitable treatment. Systematic use of the Hospital Anxiety and Depression Scale (HADS) as a rapid, clinical screening instrument could be beneficial in daily clinical practice to recognize anxiety with or without depression in MS patients.

In other words, identifying MS patients’ personality traits give us valuable information to understand how they cope with MS and personalize their therapeutic approach. The five‐factor model is the most popular, empirically supported, and scientifically useful personality taxonomy (Trull & Widiger, 2013). The five major personality dimensions are neuroticism (N), extraversion (E), openness to experience (O), agreeableness (A), and conscientiousness (C) (Trull & Widiger, 2013). Neuroticism is a tendency to experience negative emotions, such as hostility, anger, and sadness. Extraversion is described as an outgoing and sociable nature, a person who tends to be curious, imaginative, and an initiative taker. Agreeableness is a tendency to cooperate rather than compete. Conscientiousness means a lack of impulsivity, tidiness, and a goal‐oriented mind (Trull & Widiger, 2013). According to the study on 419 MS patients in 2011, the NEO‐FFI (NEO five Factor Inventory) is a reputable and validated tool for assessing MS patients’ personalities (Schwartz et al., 2011). Moreover, the primary use of this scale in MS has revealed the relationship between personality and chronic fatigue and depression (Schreiber et al., 2015).

Accordingly, we decided to evaluate the personality traits among MS patients and discover any potential correlation with anxiety and depression levels. We also aimed to investigate other potential variables that affect the severity of anxiety and depression in MS patients (disease duration and disability severity).

## 2 MATERIALS AND METHODS

### 2.1 Study design

This study is a cross‐sectional case–control study, which is performed in North Khorasan University of Medical Sciences, Iran. This study was conducted between 2019 and 2020.

### 2.2 Study population

A total of 303 individuals with a case–control ratio of 1:2 was allocated in our study. Initially, we assessed all patients who had been referred to the North‐Khorasan MS clinic with the diagnosis of MS according to MacDonald's criteria 2017, regardless of the disease type (Thompson et al., 2018). Afterward, we obtained a comprehensive medical and drug history and excluded patients with any medical conditions other than MS or consuming any medications for treatment of anxiety and depression.

Finally, we enrolled 101 patients in our study as a case group. All patients have been visited by an expert neurologist and psychologist. For each patient, we considered two healthy individuals with age and sex matched as controls. The control group included 202 individuals that were selected from students and university staff of North Khorasan University of Medical Sciences. Thorough medical and drug history was obtained from the controls in order to rule out any medical illnesses interfering with our study. Additionally, all individuals who agreed to participate signed the consent form.

The severity of disability in the MS patients was assessed based on the Expanded Disability Status Scale (EDSS), which evaluated pyramidal, cerebellar, brain stem, sensory, bowel and bladder, visual, and mental functions of patients (Şen, 2018). Patients with EDSS score 0 and 1 were considered without any disability and scores 4.5 and above were considered disabled (Lechner‐Scott et al., 2021).

### 2.3 Data collection

For all participants, demographic data and past medical and drug history were collected at the beginning of the study. The NEO‐FFI questionnaire was used to assess five major personality dimensions in all participants in both groups. This personality scale is a 60‐item self‐report questionnaire answered on a five‐point Likert scale, which ranges from 1 (strongly agree) to 5 (strongly disagree) (Baer et al., 2006).

The anxiety and depression levels of all participants in both groups were assessed through HADS. The HADS is one of the most extensively used instruments to evaluate the severity of anxiety and depression. It is sensitive and specific in recognizing pathological anxiety and has separate subscales for anxiety and depression (Giordano et al., 2011). Both scales include seven items that resulted in a total score with a possible range of 0–21. Recommended cut‐off scores are 8−10 for borderline (doubtful) cases and ≥11 for definite cases (Pais‐Ribeiro et al., 2018).

### 2.4 Sample size

In this study, we defined the sample size as all available MS patients referred to the MS clinic in North Khorasan, Iran. Accordingly, we enrolled 101 MS patients in the case group. We considered two healthy age and sex‐matched individuals for each patient as the control group (202 individuals).

### 2.5 Statistical analysis

Statistical analysis was performed by using SPSS PC Statistics (version 20.0; SPSS Inc., Chicago, IL, USA). Results have been reported as mean ± standard deviation or median (range) for normally and non‐normally distributed continuous variables, respectively. We also used numbers or percentages for nominal parameters. Kolmogorov–Smirnov test was used to assess the normality of the distribution of the variables. Independent sample *t*‐test and Mann–Whitney *U*‐test were used respectively to compare normally and non‐normally distributed variables between two groups. Fisher's exact test was used to compare proportions between the groups. We considered *p* < .05 as significant in our study.

## 3 RESULTS

The MS group consisted of 73 (73.27%) women and 27 (26.73%) men (aged 27–47 years). The control group included 149 (73.76) women and 53 (26.24) men (aged 26–45.5 years). There were no significant differences in age, sex, or annual income between the two groups (*p* = .59, *p* = .93, and *p* = .15, respectively). Educational level was almost high in control groups compared with the case group due to the social environment of this group, which were medical students and university staff. The mean period of the disease was 7.04 ± 4.9 years. Demographic characteristics of the study population are demonstrated in Table 1.

**TABLE 1:** Baseline charactristics of participants in case and control group

Parameter		Control	Case	*p* Value
Age (y) (mean ± SD)		35.54 ± 9.7	37.29 ± 9.7	.59^a^
Sex (male) %		26.24	26.73	.93^b^
Education *N* (%)	Illiterate	2 (99)	2 (1.9)	.005^*^, ^b^
	Primary	5 (2.5)	12 (11.9)	
	Secondary	82 (40.6)	46 (45.5)	
	Academic	113 (55.9)	41 (40.6)	
Average annual income *N* (%)	<400$	60 (29.7)	45 (44.5)	.15^b^
	400–800$	68 (33.7)	26 (25.7)	
	800–1600$	56 (27.7)	24 (23.7)	
	>1600$	18 (8.9)	6 (5.9)	
Mean disease duration in years (mean ± SD)			7.04 ± 4.9	
Age at onset (year) (mean ± SD)			30.25 ± 9.17	
Mean EDSS score (mean ± SD)			2.58 ± 1.59	

^a^ Independent sample *t*‐test.

^b^ Fischer's exact test.

*The significance level: p < .05.

Frequency distribution and percentages of the five dimensions scored by NEO‐FFI among MS patients at baseline are illustrated in Table 2. Based on the HADS anxiety and depression scale, most of the MS patients in our study had a score within the normal range (Table 2).

**TABLE 2:** Baseline mental status of MS patients

NEO‐FFI factors	Mean ± SD
Neuroticism	25.38 ± 7.15
Extraversion	27.6 ± 6.86
Openness to experience	24.39 ± 4.85
Agreeableness	29.58 ± 4.92
Conscientiousness	34.22 ± 5.62
HADS depression scale (%) *n*	
Normal	(64) 65
Borderline	(21) 21
Depressed	(15) 15
HADS anxiety scale (%) *n*	
Normal	(40) 40
Borderline	(27) 27
Anxious	(34) 34

Normal; HADS score ≤7, borderline (doubtful); HADS score 8−10, definite cases; HADS score ≥ 11.

The comparison of the patients’ mental status with the disease duration showed no significant difference. In other words, personality traits, depression, and anxiety levels did not differ between patients with a disease duration of ≤1 year or > 1 year (Table 3). Our study showed in patients with disease duration above 1 year, the rates of agreement (29.78%), anxiety (8.83%), and depression level (6.39%) were significantly higher than the control group (27.19, 6.47, and 4.97% respectively). Furthermore, patients with disease duration below 1 year showed a higher level of agreement and responsibility (29.65 and 34.35%, respectively) than controls (26.6 and 30.86%, respectively) (Tables S1 and S2).

**TABLE 3:** Comparison of the patients’ mental status with disease duration

NEO‐FFI factors	Disease duration ≤1y (*N* = 81)	Disease duration > 1y (*N* = 18)	95% CI	*p* Value
Neuroticism	24.89 ± 6.63	27.28 ± 9.23	−6.09 to 1.31	.2
Extraversion	27.79 ± 6.77	27.5 ± 7.5	−3.28 to 3.86	.87
Openness to experience	24.68 ± 4.88	23.28 ± 4.87	−1.12 to 3.93	.27
Agreeableness	29.65 ± 4.69	29.78 ± 6.05	−2.69 to 2.44	.92
Conscientiousness	34.35 ± 5.51	34.44 ± 5.53	−2.95 to 2.75	.95
HADS depression scale (%) n				
Normal	(66.67) 31	(61.11) 11		.21
Borderline	(17.28) 14	(33.33) 6		
Depressed	(16.05) 13	(5.56) 1		
HADS anxiety scale (%) n				
Normal	(38.27) 31	(50) 9		.49
Borderline	(29.63) 24	(16.67) 3		
Anxious	(32.1) 26	(33.33) 6		

Normal, HADS score ≤ 7; borderline (doubtful), HADS score 8−10; definite cases, HADS score≥11.

The comparison between patients with EDSS scores 0–1 and above 4.5 showed that anxiety was significantly more common in the second group. Depression was also significantly higher in patients with EDSS > 4.5 in compared with EDSS ≤ 1. Conscientiousness was the most frequent personality trait in both groups (Table 4).

**TABLE 4:** Comparison of mental status between patients with EDSS score 0–1 and >4.5

NEO‐FFI factors	EDSS score 0–1 (*N* = 15)	EDSS score > 4.5 (*N* = 14)	95% CI	*p* Value
Neuroticism	23.2 ± 7.6	26.64 ± 8.07	−9.41 to 2.53	.25
Extraversion	31.47 ± 4.47	25.36 ± 8.04	1.2 to 11.02	.02^*^
Openness to experience	24.8 ± 4.4	22.29 ± 5.51	−1.27 to 1.84	.18
Agreeableness	31.53 ± 6.33	30.64 ± 5.77	−3.74 to 2.26	.7
Conscientiousness	35.07 ± 4.83	35.5 ± 4	−3.83 to 1.65	.8
HADS depression scale (%) *n*				
Normal	(93.33) 14	(57.14) 8		.05^*^
Borderline	(6.67) 1	(14.29) 2		
Depressed	0	(28.57) 4		
HADS depression score (mean ± SD)	3.27 ± 2.49	7.5 ± 4.18	−6.84 to −1.63	.002^*^
HADS anxiety scale (%) *n*				
Normal	(46.67) 7	(28.57) 4		.08
Borderline	(46.67) 7	(28.57) 4		
Anxious	(6.67) 1	(42.86) 6		
HADS anxiety score (mean ± SD)	6.8 ± 3.23	9.64 ± 4.8	−5.94 to 0.26	.07

Normal, HADS score ≤ 7; borderline (doubtful), HADS score 8−10; definite cases, HADS score ≥ 11.

* The significance level: p < .05.

Comparing the depression and anxiety frequency between males and females revealed no significant difference between the two sexes. Moreover, the HADS scores for depression and anxiety levels were not meaningfully different between males and females (Table 5).

**TABLE 5:** Comparison of anxiety and depression prevalence between male and female patients

NEO‐FFI factors	Male (*N* = 27)	Female (*N* = 74)	95% CI	*p* Value
HADS depression scale (%) *n*				
Normal	(59.26) 16	(66.22) 49		.77
Borderline	(22.22) 6	(20.27) 15		
Depressed	(18.52) 5	(13.51) 10		
HADS depression score (mean ± SD)	6.93 ± 5.11	6.27 ± 4.33	−1.37 to 2.68	.52
HADS anxiety scale (%) *n*				
Normal	(40.74) 11	(39.19) 29		.2
Borderline	(14.81) 4	(31.08) 23		
Anxious	(44.44) 12	(29.73) 22		
HADS anxiety score (mean ± SD)	9.56 ± 6.17	8.51 ± 4.27	−1.12 to 3.2	.34

Normal, HADS score ≤ 7; borderline (doubtful), HADS score 8−10; definite cases, HADS score ≥ 11.

Comparing five factors of personality traits assessed through NEO‐FFI and depression and anxiety level between case and control groups showed that depression and anxiety HADS scores were significantly higher in the patients compared with controls. In addition, agreeableness and conscientiousness were meaningfully more common in the case group compared with the control group. The comparison between depression and anxiety HADS scores showed a significant correlation between depression and anxiety prevalence (*p* < .001, *r* = 0.65) (Table 6).

**TABLE 6:** Comparison of mental status between case and control groups

NEO‐FFI factors	Case (*N* = 101)	Control (*N* = 202)	95% CI	*p* Value
Neuroticism	25.89 ± 5.62	25.38 ± 7.15	−3.17 to −1.23	.5
Extraversion	27.08 ± 5.43	27.6 ± 6.86	−2.46 to −0.71	.47
Openness to experience	24.02 ± 4.54	24.39 ± 4.85	−1.95–0.91	.52
Agreeableness	26.7 ± 5.26	29.58 ± 4.92	−4.12 to −1.65	<.001^*^
Conscientiousness	31.01 ± 6.89	34.22 ± 5.62	−4.76 to −1.65	<.001^*^
HADS depression scale (%) *n*				
Normal	(64.36) 65	(77.72) 157		.002^*^
Borderline	(20.79) 21	(18.32) 37		
Depressed	(14.85) 15	(3.96) 8		
HADS depression score (mean ± SD)	4.86 ± 3.08	6.45 ± 4.53	−6.84 to −1.63	<.001^*^
HADS anxiety scale (%) *n*				
Normal	(39.6) 40	(64.36) 130		<.001^*^
Borderline	(26.73) 27	(18.32) 37		
Anxious	(33.66) 34	(17.33) 35		
HADS anxiety score (mean ± SD)	6.59 ± 3.6	8.79 ± 4.88	−5.94 to 0.26	<.001^*^

Normal, HADS score ≤ 7; borderline (doubtful), HADS score 8−10; definite cases, HADS score ≥ 11.

* The significance level: p < .05.

Patients with EDSS score 0–1 showed no significant difference in the prevalence of depression and anxiety compared with control groups (*p* = .24 vs. .65, respectively). The comparison between patients with EDSS score >4.5 and the controls represented a significant difference in the prevalence of depression and anxiety compared with control groups (*p* = .05 and .03, respectively) (Table 7).

**TABLE 7:** Comparison of mental status between case and cotrol groups in patients with EDSS score 0–1 and EDSS >4.5

NEO‐FFI factors	Case (*N* = 101) (mean ± SD)	Control (*N* = 202) (mean ± SD)	95% CI	*p* Value
Neuroticism		25.9 ± 5.66		
EDSS score 0–1	23.2 ± 7.6		1.343	.186
EDSS > 4.5	26.64 ± 8.07		−0.631	.532
Extraversion		26.5 ± 5.84		
EDSS score 0–1	31.47 ± 4.47		−2.891	.006^*^
EDSS > 4.5	25.36 ± 8.03		1.234	.224
Openness to experience		24.4 ± 4.9		
EDSS score 0–1	24.8 ± 4.4		−0.267	.791
EDSS > 4.5	22.29 ± 5.51		0.561	.578
Agreeableness		26.23 ± 4.97		
EDSS score 0–1	31.53 ± 6.33		−3.073	.004^*^
EDSS > 4.5	30.64 ± 5.77		2.464	.018^*^
Conscientiousness		30.33 ± 6.91		
EDSS score 0–1	35.07 ± 4.83		−2.371	.022^*^
EDSS > 4.5	35.5 ± 4		2.812	.008^*^
HADS depression (mean ± SD)		3.67 ± 2.86		
EDSS score 0–1	3.27 ± 2.49		0.461	.647
EDSS > 4.5	7.5 ± 4.18		−2.268	.029*
HADS anxiety score (mean ± SD)		5.6 ± 3.16		
EDSS score 0–1	6.8 ± 3.23		−1.192	.240
EDSS > 4.5	9.64 ± 4.8		−2.005	.052^*^

NEO‐FFI, Neuroticism‐Extraversion‐Openness Five‐Factor Inventory; EDSS, Expanded Disability Status Scale; HADS, Hospital Anxiety and Depression Scale.

* The significance level: p < .05.

## 4 DISCUSSION

In this study, we have compared the mental status (personality traits, depression, and anxiety levels) of 101 MS patients with 202 controls.

Evidently, the prevalence of depression in MS patients (1 year and lifelong prevalence rate of 20% and 40–50%, respectively) is considerably higher than in the general population or among patients with chronic medical conditions other than MS (Alsaadi et al., 2015; Skokou et al., 2012).

In our study, about 15% of patients experienced depression based on the HADS depression scale and this rate was higher in the first year of MS diagnosis. Our result is in accordance with a previous study in this regard (Possa et al., 2017). Such that, newly diagnosed patients suffered from psychological changes immediately after diagnosis. This can not only affect the patients’ QoL but also decrease medication adherence and compliance.

In our study, the mean HADS depression score was higher than a similar study (6.45 vs. 3.8, respectively) (Janssens et al., 2003). Some methodological differences should be considered before interpreting these findings as follow: (1) our study recruited patients from MS clinics, therefore, patients coping well in the community might be underreported; (2) although there are various criteria and guidelines for depression diagnosis in MS patients, there is little or no consensus regarding clinical “gold standard” for diagnosing depression; (3) some somatic symptoms of MS‐like fatigue, might be misdiagnosed as depression, mainly if behavioral rating scales were used; and (4) the number of patients in the first year of diagnosis in this study was much larger than patients with disease duration above 1 year, which makes the judgment difficult.

Our study revealed a significantly higher rate of depression among patients with EDSS scores of 4.5 and above. In some previous studies, the relationship between depression and disability levels among MS patients has been reported, while it is still controversial (Amato et al., 2001; da Silva et al., 2011; Kroencke et al., 2000).

In our study, the prevalence of depression was not meaningfully different between the two sexes. In other words, there is no correlation between depression and sex among the MS population, which was consistent with some previous studies in this regard (Beiske et al., 2008; Karimi et al., 2020; Kessler & Bromet, 2013). This result is not similar to the prevalence of depression in the general population. Depression is more common in women. Therefore, the neurological illness among MS patients may contribute to the core pathophysiology of depression regardless of sex (Beiske et al., 2008).

In our study, depression was significantly higher in the MS group than in the control group. This agrees with a similar previous study that reported depression in 31.4% of MS patients. It is twice higher rate than the general population (Beiske et al., 2008).

Anxiety is another frequent psychological problem in MS patients. Our study showed that anxiety occurred in 33.66% of patients based on HADS score, which was significantly higher than the control group. Beiske et al. (2008) reported anxiety occurs in 19.3% of MS patients, which is three times higher than the general population.

Anxiety can occur coincidentally in many patients with depression (Wood et al., 2013). They cause disabling problems that affect the general health and the QoL in MS patients. In our study, 23 patients out of 101 cases experienced depression and anxiety concomitantly based on the HADS scale. This is in accordance with the Smith and Young study that reported 34% coincidence of depression and anxiety and also showed depression powerfully predicted anxiety. Anxiety, in turn, strongly predicted later depression (Smith & Young, 2000). The Garfield & Lincoln (2012) study also found depression is the most significant factor related to anxiety.

Our study showed the high rates of anxiety among MS patients’ parents and partners than the control group (27.2 vs. 17.33%, respectively, *p* = .012). Janssens et al. (2006) also assessed 101 newly diagnosed MS patients and their partners and found high rates of anxiety among their partners (40%).

Additionally, our study revealed patients with more functional limitations (EDSS > 4.5) had significantly higher levels of anxiety and depression than controls, which is in line with previous findings in EDSS ≥ 3 (Janssens et al., 2006). Conversely, the Dahl et al. (2009) case–control study on 172 MS patients showed no significant difference in the EDSS score between the different levels of anxiety or depression.

Because of the high coincidence of depression and anxiety among the MS population, an anxiety disorder in this group is under‐treatment. Moreover, depression affected their ability to engage in treatment and consequently is affected their QoL. Considering cognitive‐behavioral therapy‐based treatment could be more helpful in these patients rather than only targeting the treatment plan on curing specific MS symptoms.

The most frequent dimension seen in the MS group was conscientiousness, which means that these patients lack impulsivity. We also found that this trait significantly is higher in the MS group than controls (35 vs. 30%, respectively, *p* = .022). Our findings are in line with the Lima et al. (2015) study in Brazil. Conversely, Hawkes (2005) found a somewhat different finding while examining impulsiveness or risk‐disregarding behavior in MS patients. According to Hawkes's study, MS patients neglected healthy behaviors compared with healthy controls (Hawkes, 2005). For example, higher smoking rates and alcohol consumption were reported in this population. Another study similarly found less conscientiousness in MS patients (Benedict et al., 2001). They proposed that patients with low conscientiousness were at a higher risk for developing MS‐related neuropsychiatric symptoms.

Agreeableness was the second most frequent trait found in this group. Agreeable people are more cooperative than competitive. Other studies on the personality of MS patients found a similar pattern (Benedict et al., 2001). Generally, if this trait is due to confrontation avoidance, agreeable people can have low self‐esteem. This trait also was significantly higher in MS patients than in the general population (31 vs. 26%, respectively, *p* = .004).

Our study suffered from some limitations merit consideration. First, our study was designed as a cross‐sectional study without prospective evidence. Therefore, finding the causal relationships between evidence is more difficult. Second, we used the HADS scale for assessing depression and anxiety, which is a self‐report measure. We suggest more studies with a larger population. We also suggest using the formal Diagnostic and Statistical Manual of Mental Disorders, fifth edition (DSM‐V) scale for diagnosing in order to fund more reliable results compared with the self‐report scale.

## 5 CONCLUSIONS

Anxiety and depression levels (based on HADS score) were much higher in the patients with a high disability index (above 4.5) than patients with a low disability index (below 1). This can be considered an influential factor on the MS patients’ mental health and QoL, especially during the first year after diagnosing. Anxiety and depression levels were also higher in the MS patients compared with the controls. The results of our study showed that the level of conscientiousness and agreement in patients was higher than in healthy individuals.

Accordingly, MS patients suffered from psychological changes from the initial stages of the disease. Therefore, a complete comprehension of these conditions by the neurologist could be vital in improving patients’ QoL and increasing patients’ compliance and adherence to pharmacological therapy.

## FUNDING INFORMATION

This article was sponsored by the Research Council of Bojnord University of Medical Sciences (Bojnord, North‐Khorasan, Iran). The funder of the study had no role in study design, data collection, data analysis, data interpretation, or writing of the report.

## CONFLICT OF INTEREST

We declare no competing interests, financial or otherwise.

### PEER REVIEW

The peer review history for this article is available at https://publons.com/publon/10.1002/brb3.2596.

## Supporting information

Table S1 Comparison of the patients’ mental status with disease duration ≤ 1 year
Table S2 Comparison of the patients’ mental status with disease duration > 1 year

## Data Availability

The data that support the findings of this study are available upon reasonable request from the corresponding author. The data are not publicly available due to privacy or ethical restrictions.
