# Supplementary material for: Personality traits of patients with multiple sclerosis and their correlation with anxiety and depression levels: A cross‐sectional case–control study
Source: Brain Behav. 2022 Apr 22;12(5):e2596. doi: 10.1002/brb3.2596 (PMC9120902; doi:10.1002/brb3.2596)
Supplement: Supplementary file 1 — Table S1 Comparison of the patients’ mental status with disease duration ≤ 1 year Table S2 Comparison of the patients’ mental status with disease duration > 1 year [file BRB3-12-e2596-s001.docx]

Supplement 1 Comparison of the patients’ mental status with disease duration ≤ 1 year

| NEO-FFI factors | Case (N=81)  (Mean±SD) | Control (N=162)  (Mean±SD) | 95% CI | P value |
| --- | --- | --- | --- | --- |
| Neuroticism | 24.89(6.63) | 26.14(5.64) | 1.538 | .125 |
| Extraversion | 27.79(6.77) | 26.96(5.52) | - 1.020 | .309 |
| Openness to experience | 24.68(4.88) | 23.98(4.48) | - 1.110 | .268 |
| Agreeableness | 29.65(4.69) | 26.6(5.42) | -4.531 | ^*^< .001 |
| Conscientiousness | 34.35(5.51) | 30.86(6.83) | - 4.278 | ^*^< .001 |
| HADS depression score (mean±SD) | 8.58(4.64) | 6.64(3.63) | -3.290 | ^*^.001 |
| HADS anxiety score  (mean±SD) | 6.3(4.61) | 4.97(3.1) | -2.341 | .021 |

NEO-FFI, Neuroticism-Extraversion-Openness Five-Factor Inventory; HADS, Hospital Anxiety and Depression Scale.

*The significance level: p < .05

Supplement 2 Comparison of the patients’ mental status with disease duration > 1 year

| NEO-FFI factors | Case (N=18)  (Mean±SD) | Control (N=36)  (Mean±SD) | 95% CI | P value |
| --- | --- | --- | --- | --- |
| Neuroticism | 27.28 (9.23) | 24.89 (5.7) | - 1.006 | .246 |
| Extraversion | 27.5 (7.5) | 27.89 (5.15) | .224 | .824 |
| Openness to experience | 23.28 (4.87) | 23.64 (4.67) | .264 | .793 |
| Agreeableness | 29.78 (6.05) | 27.19 (4.61) | - 1.746 | .087 |
| Conscientiousness | 34.44 (5.53) | 31.94 (7.07) | - 1.310 | .196 |
| HADS depression score (mean±SD) | 8.83 (5.31) | 6.47 (3.66) | - 1.916 | .061 |
| HADS anxiety score  (mean±SD) | 6.39 (3.73) | 4.61 (3.13) | -1.846 | .071 |

NEO-FFI, Neuroticism-Extraversion-Openness Five-Factor Inventory; HADS, Hospital Anxiety and Depression Scale.
